# Pro‐ and anti‐inflammatory cytokines mediate the progression of severe anemia in malaria‐infected children: A prospective study

**DOI:** 10.1002/iid3.70013

**Published:** 2024-09-06

**Authors:** Charles Nkansah, Felix Osei‐Boakye, Gabriel Abbam, Samuel K. Appiah, Samira Daud, Bright Boakye, Samsiyatu Abdulai, Madina Ahmed, Theophilus B. Antwi, Birago Boateng, Miigbat P. Libatin, Alexander S. Mensah, Mary K. Missah, Richard V. Duneeh, Ashiya Haruna, Stephany Adda, Pagnaa G. Abdul‐Rauf, Zacharia A. Ofori, George B. Fosu, Sandra Segnitome, Isaac Adjei, Emmanuel Appiah‐Kubi, Moses Banyeh, Charles A. Derigubah, Muniru M. Tanko, Ejike F. Chukwurah

**Affiliations:** ^1^ Department of Haematology, School of Allied Health Sciences University for Development Studies Tamale Ghana; ^2^ Department of Medical Laboratory Science, Faculty of Health Sciences and Technology Ebonyi State University Abakaliki Nigeria; ^3^ Department of Medical Laboratory Technology, Faculty of Applied Science and Technology Sunyani Technical University Sunyani Ghana; ^4^ Department of Biomedical Laboratory Sciences, School of Allied Health Sciences University for Development Studies Tamale Ghana; ^5^ Department of Medical Laboratory Science, School of Allied Health Sciences University of Health and Allied Sciences Ho Ghana; ^6^ Haematology Unit, Department of Medical Laboratory Tamale Teaching Hospital Tamale Ghana; ^7^ Department of Medical Laboratory Technology, School of Applied Science and Arts Bolgatanga Technical University Bolgatanga Ghana

**Keywords:** children, cytokines, malaria, *P. falciparum*, severe malarial anemia

## Abstract

**Background:**

Severe *Plasmodium falciparum* malarial anemia is still the principal cause of death in children in underdeveloped countries. An imbalance between proinflammatory and anti‐inflammatory cytokines is associated with malaria progression. This study evaluated circulating levels of selected inflammatory cytokines among malaria‐infected children in Ghana.

**Methods:**

This case‐control study was conducted at Tamale Teaching Hospital, Ghana. One hundred and twenty children with malaria and 60 controls, aged 12−144 months were selected from April to July, 2023 for the study. Malaria was diagnosed through microscopy, full blood count was measured using hematology analyzer, and cytokines were measured using enzyme‐linked immunosorbent assay.

**Results:**

Malaria‐infected children had higher tumor necrosis factor alpha (TNF‐α) (*p* < .001), interferon‐gamma (IFN‐ɣ) (*p* < .001), interleukin (IL)‐1β (*p* < .001), IL‐6 (*p* < .001), granulocyte macrophage‐colony stimulating factor (GM‐CSF) (*p* < .001), and IL‐10 (*p* < .001) levels than controls. Participants with high parasitemia had raised TNF‐α (*p* < .001), IFN‐ɣ (*p* < .001), IL‐1β (*p* < .001), IL‐6 (*p* < .001), GM‐CSF (*p* < .001), and IL‐10 (*p* < .001), but reduced IL‐3 (*p* < .001) and TGF‐β (*p* < .001) than those with low parasitemia. Severe malarial anemic children had elevated TNF‐α (*p* < .001), IFN‐ɣ (*p* < .001), IL‐1β (*p* < .001), IL‐6 (*p* < .001), GM‐CSF (*p* < .001), and IL‐10 (*p* < .001), but lower IL‐3 (*p* < .001) and TGF‐β (*p* < .001) than those with uncomplicated malaria.

**Conclusion:**

Parasite density was the principal predictor of the cytokine levels, as parasitemia positively associated with IL‐10, GM‐CSF, IL‐6, IL‐1β, IFN‐ɣ, and TNF‐α, but negatively associated with IL‐3 and TGF‐β. Malaria is associated with enhanced secretion of pro‐ and anti‐inflammatory cytokines in Ghanaian children. Inflammatory cytokines may be involved in the development of severe malarial anemia in children. However, IL‐3 and TGF‐β may offer protection against severe malarial anemia.

## 1 INTRODUCTION

Ghana is one of the developing countries heavily affected by malaria, and the situation worsens during the rainy season when mosquito bites are on the rise. The extensive interventions implemented over the years to control malaria in Ghana have had tremendous positive effects, with national prevalence in children declining by more than one‐third from 26.7% in 2014 to 8.6% in 2022.^1^ Despite this milestone in malaria control and prevention, malaria is still the principal cause of morbidity and mortality in Ghana, with children less than 5 years of age extremely affected.^2^ In Tamale, Northern Ghana, where malaria is holoendemic, *Plasmodium falciparum* is the principal cause of hematological and neurological disorders, and the leading contributor of hospitalization, especially in children.^3^

Anemia is the most common complication of malaria in the tropics, and results from the related extensive sequestration of infected erythrocytes, lysis of erythrocytes, suppression of bone marrow, dyserythropoiesis, complement mediated, renal suppression of erythropoietin secretion, and excessive inflammatory response.^4^, ^5^ The inoculation of plasmodium sporozoites into the subcutaneous layer of the human host and the associated complex life cycle of the parasite stimulate both innate and adaptive immunity responses.^6^, ^7^ Malaria‐infected erythrocytes express *P. falciparum* erythrocyte membrane protein‐1 (*Pf*EMP‐1), which enhances splenic and bone marrow sequestration of both parasitized and non‐parasitized cells. Frequent sequestration of the cells in various organs disrupts the endothelium, triggers inflammation to release inflammatory cytokines. Proinflammatory cytokines contribute significantly to malaria protection and parasite elimination.^7^, ^8^ During acute malaria, there is immediate secretion of pro‐inflammatory T helper 1 (Th1) cytokines such as interleukin (IL)−1β, IL‐6, interferon‐gamma (IFN‐ɣ) and tumor necrosis factor alpha (TNF‐α), which retard parasite replication, enhance monocyte phagocytosis and promote elimination of infected red blood cells (RBCs).^6^ This tends to prevent the advancement from uncomplicated malaria to severe malaria and to prevent life‐threatening complications. Elevated levels of proinflammatory cytokines have been reported among malaria‐infected children in previous studies.^4^, ^9^, ^10^, ^11^, ^12^ Th17 cells‐mediated proinflammatory cytokines including IL‐17 and IL‐22 also play significant roles in immune response following plasmodium infection, by recruiting neutrophils, and triggering the release of other proinflammatory cytokines.^11^ Earlier studies in Ghana and Gabon reported increased IL‐17 and IL‐22 levels among children with plasmodium malaria.^11^, ^12^ However, excess release of these proinflammatory cytokines during acute malaria may be detrimental to the host and cause severe complications such as severe anemia, organ damage and death.^6^ To modulate the secretion of proinflammatory cytokines and their associated complications in malaria, Th2 cell‐mediated anti‐inflammatory cytokines such as IL‐10, IL‐4, IL‐13, and transforming growth factor‐beta (TGF‐β) are produced. Anti‐inflammatory cytokines control the humoral immunity, promoting parasite replication and clearance, and retard Th1 cytokine secretion.^13^ Disequilibrium between proinflammatory and anti‐inflammatory cytokines results in noticeable adverse complications which are linked to malaria severity and an increased mortality rate especially in children.^13^ Hence, there is a need for a proinflammatory and anti‐inflammatory balance to avert life‐threatening complications associated with malaria.

Regardless of the clinical significance of pro‐ and anti‐inflammatory cytokines in the host immune response against *P. falciparum* malaria, there is limited data among children in Northern Ghana. Therefore, this study assessed selected pro‐ and anti‐inflammatory cytokines among malaria‐infected children in Northern Ghana. The findings from this study will be beneficial in the management of severe malarial anemia in children.

## 2 MATERIALS AND METHODS

### 2.1 Ethical consideration and informed consent

This study was conducted in accordance with the Declaration of Helsinki on Research involving Humans. The Institutional Review Board of University for Development Studies, Ghana approved this study (UDS/IRB/15/2023). Permission was gotten from Tamale Teaching Hospital. Written informed consent was obtained from the parents or guardians of the participants by signing or thumb‐printing the written informed consent form.

### 2.2 Study participants

One hundred and twenty malaria‐infected children, aged 12−144 months were recruited from April to July, 2023 at Tamale Teaching Hospital, Tamale, Ghana. Sixty apparently healthy age‐ and sex‐matched children without malaria were selected from a Basic School in Tamale as controls. Tamale is the capital of the Northern Region, with a population of about 371,351 and is located at latitude 9.3930 N and longitude 0.8235 W. The hospital's digital address is NT‐0101‐5777. *P. falciparum* transmission is holoendemic and remains a major cause of morbidity and mortality in inhabitants of the area, with children being the most vulnerable.^3^ Major malaria transmission in Tamale occurs in the rainy season (April to June), and this area has benefited from the Seasonal Malaria Chemoprevention program implemented by the National Malaria Control Program, Ghana since 2015. This case‐control study used Kelsey's formulae to determine sample size. Malaria parasitemia was graded into low parasite density (<1000 parasites/µL), moderate parasite density (1000−10,000 parasites/µL) and high parasite density (>10,000 parasites/µL).^11^ Participants were further categorized based on the severity of anemia as mild/moderate malarial anemia (presence of malaria parasite and Hb ≥ 5.0 < 11.0 g/dL), and severe malarial anemia (presence of malaria parasite and Hb<5.0 g/dL).^14^ Age, sex and axillary temperature were collected from the participants' clinical records. Exclusion criteria included participants receiving anti‐malarial drugs, comorbid with hemoglobinopathies, glucose‐6‐phosphate dehydrogenase deficiencies, human immunodeficiency virus, helminth infection, and any other disease that may promote inflammation or influence hematological parameters. Participants whose parents or guardians withheld their consent were excluded from the study.

### 2.3 Laboratory assays

Thick and thin blood films were prepared from 6 and 2 µL, of dipotassium ethylenediaminetetraacetic acid (K_2_EDTA)‐anticoagulated whole blood collected from each participant, respectively. The films were stained with 10% Giemsa working solution, examined under light microscope (Olympus) by two microscopy experts, and parasitemia was evaluated by multiplying the parasite count by the absolute leucocytes, and dividing by a set range of leukocytes (≥200 leukocytes).^14^ Full blood count (FBC) was measured using an automated hematology analyzer (URIT‐5250, China). Malaria parasite identification and quantification, as well as FBC measurements, were performed immediately, and samples were collected daily at the Hematology Laboratory of the Tamale Teaching Hospital. To preserve bio‐active TGF‐β, platelet‐poor plasma was collected from the EDTA‐anticoagulated blood on ice. The blood was centrifuged at 1000*g* for 15 min within 30 min of sample collection. Further centrifugation of the extracted plasma at 10,000*g* for 10 min at 4°C was done to ensure complete removal of platelet. Platelet count in the plasma was <10.0 × 10^9^/L for quality control purposes. The platelet‐poor plasma was aliquoted, and stored at −20°C until analysis. Plasma levels of proinflammatory cytokines: TNF‐α, IFN‐ɣ, IL‐1β, IL‐6, granulocyte macrophage‐colony stimulating factor (GM‐CSF), and IL‐3, and anti‐inflammatory cytokines: IL‐10 and TGF‐β were measured using sandwich enzyme‐linked immunosorbent assay, with commercially prepared reagents (Biobase) at the University for Development Studies Laboratory, in accordance with manufacturer's instructions. The absorbance and corresponding concentrations of the cytokines were read at 450 nm using automated microplate reader (Poweam).

### 2.4 Data analysis

IBM SPSS software version 26.0 was used to analyze the data. Normally distributed data were presented as mean ± standard deviation (SD), and skewed data were presented as median (1^st^−3^rd^ quartiles). Categorical data were presented as frequencies with corresponding percentages, and compared using Pearson Chi‐square. Continuous data between two categories were compared using Independent Student's *T* test (for parametric data) and Mann−Whitney *U* test (for non‐parametric data). Plasma levels of inflammatory cytokines within three categories (parasitemia grading and anemia severity) were compared using the Kruskal−Wallis test. Multiple linear regression was used to examine age, sex, malaria status, parasite density and anemia severity for predictors of cytokine levels. Statistical significance was set at *p* < .05.

## 3 RESULTS

### 3.1 Demographic, clinical, and hematological characteristics of the study participants

One hundred and twenty out of the 180 participants had the asexual form of *P. falciparum* in peripheral blood, and 60 were uninfected. The median age of the plasmodium‐infected children and the controls were 24.0 (12.0−81.0) and 36.0 (12.0−84.0) months, respectively, but this was not statistically significant. About two‐thirds of the participants were younger than 60 months with male percentage of 58.9%. The median parasite density was 440.0 (96.0−34,451.0) parasites/µL.

Of the 120 *P. falciparum*‐infected children, the majority (56.7%) had low parasitemia in peripheral blood, and 41.7% had severe anemia. Children infected with *P. falciparum* had significantly lower RBC (*p* < .001), Hb (*p* < .001), HCT (*p* < .001), lymphocyte counts (*p* = .004), and platelet counts (*p* < .001), but higher neutrophil counts (*p* = .021) than uninfected controls (Table 1).

**Table 1:** Demographic, clinical and hematological characteristics of the study participants.

Variables	Study participants		*p* Value
	Malaria‐infected (*N* = 120)	Uninfected controls (*N* = 60)	
*Demographics*			
Age (months)	24.0 (12.0−81.0)	36.0 (12.0−84.0)	.800
*Age category*			.512
<60	74 (61.7)	40 (66.7)	
60−144	46 (38.3)	20 (33.3)	
*Sex*			.391
Males	68 (56.7)	38 (63.3)	
Females	52 (43.3)	22 (36.7)	
*Clinical characteristics*			
Parasite density (p/µL)	440.0 (96.0−34451.0)	NA	NA
*Degree of parasitemia (p/µL)*			
<1000	68 (56.7)	NA	NA
1000−10,000	17 (14.2)	NA	NA
>10000	35 (29.2)	NA	NA
*Anemia severity*			
Malaria only	20 (16.6)	NA	NA
Mild/moderate malarial anemia	50 (41.7)	NA	NA
Severe malarial anemia	50 (41.7)	NA	NA
*Blood cell parameters*			
RBC × $10^{6}$/µL	3.3 (1.5−3.6)	4.0 (3.9−4.5)	<.001
Hb (g/dL)	9.4 (4.7−10.7)	11.9 (11.4−12.5)	<.001
HCT%	27.8 (14.4−31.7)	35.6 (34.6−36.8)	<.001
MCV (fL)	78.4 (68.5−81.7)	77.0 (66.2−83.9)	.903
MCH (pg)	26.4 ± 4.4	24.7 ± 4.8	.669
MCHC (g/dL)	34.2 ± 3.3	34.0 ± 2.1	.586
RDW‐CV%	10.1 (8.5−11.9)	9.0 (8.1−11.5)	.097
WBC × $10^{3}$/µL	9.0 (6.0−12.6)	9.9 (7.0−13.9)	.652
Neut. (%)	56.5 (41.6−67.7)	44.1 (36.2−63.1)	.021
Lymph. (%)	28.6 (21.3−43.4)	41.5 (23.1−53.7)	.004
Mono. (%)	7.4 (5.6−11.6)	7.3 (5.0−9.0)	.341
Eos. (%)	1.8 (0.6−3.8)	1.5 (0.7−4.8)	.636
Baso. (%)	0.08 (0.06−0.14)	0.08 (0.07−0.12)	.810
PLT × $10^{3}$/µL	205.5 (133.3−304.5)	9.9 (7.0−13.9)	<.001

*Note*: Categorical data were presented as frequencies with corresponding percentages, and compared using Pearson chi‐square test. Parametric data are presented as mean ± SD, and compared using Student's *t*‐test. Nonparametric data were presented in medians with corresponding interquartile ranges, and compared using Mann Whitney *U* test. Statistical significance was set at *p* < .05.

Abbreviations: Baso., basophils; Eos., eosinophils; fL, femtolitre; g/dL, grams per deciliter; Hb, hemoglobin concentration; HCT, hematocrit; Lymph., lymphocytes; MCH, mean cell hemoglobin; MCHC, mean cell hemoglobin concentration; MCV, mean cell volume; Mono., Monocytes; *N*, number of participants; Neut., neutrophils; p/µL, parasites per microlitre; pg, picogram; PLT, platelet count; RBC, absolute red blood cell count; RDW‐CV, red blood cell distribution width‐coefficient of variation; WBC, white blood cell count; µL, microlitre.

### 3.2 Inflammatory cytokines between *P. falciparum*‐infected children and controls

Malaria‐infected children had relatively higher TNF‐α (*p* < .001), IFN‐ɣ (*p* < .001), IL‐1β (*p* < .001), IL‐6 (*p* < .001), GM‐CSF (*p* < .001), and IL‐10 (*p* < .001) levels than uninfected participants. However, the plasma levels of IL‐3 and TGF‐β were indifferent between cases and controls. Participants with malaria had higher TNF‐α/IL‐10, TNF‐α/TGF‐β, IFN‐ɣ/TGF‐β, IL‐1β/TGF‐β and, but lower IFN‐ɣ/IL‐10, IL‐1β/IL‐10, and IL‐6/TGF‐β ratios than those in the control group (Table 2).

**Table 2:** Inflammatory cytokines between malaria‐infected children and controls.

Inflammatory cytokines	Study participants		*p* Value
	Malaria‐infected (*N* = 120)	Uninfected controls (*N* = 60)	
***Proinflammatory cytokines***			
TNF‐α (pg/mL)	54.2 (22.0‐83.5)	4.4 (3.9‐5.8)	<.001
IFN‐ɣ (pg/mL)	147.5 (121.3‐306.8)	85.4 (61.0‐96.0)	<.001
IL‐1β (pg/mL)	33.7 (18.5‐57.9)	12.1 (10.0‐14.0)	<.001
IL‐6 (ng/L)	68.2 (51.8‐123.7)	14.9 (13.2‐17.6)	<.001
GM‐CSF (ng/L)	24.1 (18.2‐38.0)	12.9 (9.9‐16.0)	<.001
IL‐3 (pg/mL)	6.9 (5.7‐8.2)	6.6 (6.1‐7.1)	.372
*Anti‐inflammatory cytokines*			
IL‐10 (ng/L)	107.4 (60.5‐329.3)	22.3 (15.0‐28.7)	<.001
TGF‐β (pg/mL)	1986.9 (962.8‐2946.8)	1938.0 (1420.4‐2277.0)	.961
*Cytokine ratios*			
TNF‐α/IL‐10	0.3 (0.2‐0.5)	0.2 (0.1‐0.4)	<.001
IFN‐ɣ/IL‐10	1.3 (0.9‐2.1)	3.9 (2.0‐6.4)	<.001
IL‐Iβ/IL‐10	0.3 (0.2‐0.3)	0.6 (0.3‐0.9)	<.001
IL‐6/IL‐10	0.6 (0.5‐1.0)	0.7 (0.4‐1.2)	.369
TNF‐α/TGF‐β	0.02 (0.01‐0.09)	0.002 (0.002‐0.004)	<.001
IFN‐ɣ/TGF‐β	0.1 (0.04‐0.3)	0.05 (0.03‐0.07)	<.001
IL‐Iβ/TGF‐β	0.02 (0.01‐0.06)	0.01 (0.0‐0.01)	<.001
IL‐6/TGF‐β	0.03 (0.02‐0.14)	0.01 (0.0‐0.01)	<.001

*Note*: The data are presented as median (interquartile range), and compared using the Mann‐Whitney U test. p < .05 was considered statistically significant.

Abbreviations: GM‐CSF, granulocyte macrophage‐colony stimulating factor; IFN‐ɣ, interferon‐gamma; IL, interleukin; *N*, number of participants; ng/L, nanogram per liter; pg/mL, Picogram per milliliter; TGF‐β, transforming growth factor‐beta; TNF‐α, tumor necrosis factor‐alpha.

### 3.3 Age‐specific pro‐ and anti‐inflammatory cytokines of the study participants

Among both children less than 5 years, and those from 5 to 12 years, TNF‐α (*p* < .001), IFN‐ɣ (*p* < .001), IL‐1β (*p* < .001), IL‐6 (*p* < .001), GM‐CSF (*p* < .001), and IL‐10 (*p* < .001) levels were elevated in the participants with malaria compared with the apparently healthy children without malaria (Table 3).

**Table 3:** Age‐specific pro‐ and anti‐inflammatory cytokines of the study participants.

Inflammatory cytokines	Ages of participants					
	<60 months (*N* = 114)			60−144 months (*N* = 66)		
	Cases (74)	Controls (40)	*p* Value	Cases (46)	Controls (20)	*p* Value
*Proinflammatory cytokines*						
TNF‐α (pg/mL)	46.1 (24.0‐74.0)	4.3 (3.8‐7.7)	<.001	61.2 (20.6‐88.1)	5.2 (4.0‐5.8)	<.001
IFN‐ɣ (pg/mL)	146.0 (118.0‐291.5)	85.4 (59.5‐96.8)	<.001	164.0 (123.0‐329.5)	85.5 (68.0‐91.0)	<.001
IL‐1β (pg/mL)	29.1 (18.0‐52.3)	12.0 (9.8‐13.8	<.001	36.0 (22.0‐60.9)	12.9 (10.5‐14.2)	<.001
IL‐6 (ng/L)	68.4 (52.7‐117.8)	14.7 (13.0‐17.9)	<.001	68.0 (51.0‐124.6)	16.1 (13.4‐16.8)	<.001
GM‐CSF (ng/L)	23.0 (17.9‐37.3)	12.7 (9.8‐16.9)	<.001	27.0 (19.0‐45.9)	12.9 (9.9‐14.0)	<.001
IL‐3 (pg/mL)	6.8 (6.0‐8.3)	6.6 (5.8‐7.5)	.354	7.0 (4.7‐8.0)	6.6 (6.2‐6.8)	.823
*Anti‐inflammatory cytokines*						
IL‐10 (ng/L)	89.0 (55.8‐267.0)	23.0 (14.9‐31.0)	<.001	134.0 (67.0‐398.7)	18.7 (16.2‐25.4)	<.001
TGF‐β (pg/mL)	2120.0 (978.3‐3061.9)	1988.4 (1432.6‐2504.8)	.585	1877.0 (844.0‐2589.0)	1673.9 (1398.0‐2254.0)	.577

*Note*: The data are presented as median (interquartile range), and compared using the Mann−Whitney *U* test. *p* < .05 was considered statistically significant.

Abbreviations: GM‐CSF, granulocyte macrophage‐colony stimulating factor; IFN‐ɣ, interferon‐gamma; IL, interleukin; *N*, number of participants; ng/L, nanogram per liter; pg/mL, picogram per milliliter; TGF‐β, transforming growth factor‐beta; TNF‐α, tumor necrosis factor‐alpha.

### 3.4 Sex‐specific pro‐ and anti‐inflammatory cytokines of the study participants

The proinflammatory cytokines: TNF‐α (*p* < .001), IFN‐ɣ (*p* < .001), IL‐1β (*p* < .001), IL‐6 (*p* < .001), GM‐CSF (*p* < .001), and the anti‐inflammatory cytokine IL‐10 (*p* < .001) levels were significantly higher in malaria‐infected males and females than in their counterparts without malaria (Table 4).

**Table 4:** Sex‐specific pro‐ and anti‐inflammatory cytokines of the study participants.

Inflammatory cytokines	Sex of participants					
	Males (*N* = 106)			Females (*N* = 74)		
	Cases (68)	Controls (38)	*p* Value	Cases (52)	Controls (22)	*p* Value
*Proinflammatory cytokines*						
TNF‐α (pg/mL)	63.0 (36.0‐82.1)	4.3 (3.9‐5.5)	<.001	39.9 (17.0‐86.2)	5.0 (3.2‐8.4)	<.001
IFN‐ɣ (pg/mL)	233.5 (131.0‐303.0)	86.7 (65.0‐96.0)	<.001	132.0 (107.0‐312.2)	84.0 (46.0‐96.0)	<.001
IL‐1β (pg/mL)	35.4 (21.6‐58.7)	12.0 (10.5‐13.0)	<.001	27.0 (16.2‐55.0)	12.8 (9.4‐14.3)	<.001
IL‐6 (ng/L)	73.1 (61.5‐113.7)	14.8 (13.4‐16.8)	<.001	65.1 (39.0‐142.0)	16.2 (7.4‐18.0)	<.001
GM‐CSF (ng/L)	27.9 (21.0‐38.0)	13.5 (9.7‐17.0)	<.001	20.7 (14.8‐38.0)	11.0 (9.9‐15.0)	<.001
IL‐3 (pg/mL)	6.9 (5.7‐8.3)	6.6 (6.1‐6.8)	.322	6.9 (6.0‐8.0)	6.5 (5.7‐7.9)	.723
*Anti‐inflammatory cytokines*						
IL‐10 (ng/L)	129.0 (69.0‐315.0)	23.0 (15.9‐27.0)	<.001	69.9 (51.4‐345.0)	18.2 (14.8‐34.0)	<.001
TGF‐β (pg/mL)	1659.5 (986.0‐2660.0)	1986.0 (1469.0‐2254.0)	.292	2295.5 (923.0‐3160.4)	1633.7 (1287.0‐2523.0)	.276

*Note*: The data are presented as median (interquartile range), and compared using the Mann−Whitney *U* test. *p* < 0.05 was considered statistically significant.

Abbreviations: GM‐CSF, granulocyte macrophage‐colony stimulating factor; IFN‐ɣ, interferon‐gamma; IL, interleukin; *N*, number of participants; ng/L, nanogram per liter; pg/mL, picogram per milliliter; TGF‐β, transforming growth factor‐beta; TNF‐α, tumor necrosis factor‐alpha.

### 3.5 Relationship between inflammatory cytokines and degree of parasitemia among malaria‐infected participants

There was a significant relationship between inflammatory cytokines and malaria parasitemia. Children with high parasite density (>10,000 parasites/µL) had higher plasma levels of TNF‐α (*p* < .001), IFN‐ɣ (*p* < .001), IL‐1β (*p* < .001), IL‐6 (*p* < .001), GM‐CSF (*p* < .001), and IL‐10 (*p* < .001) than those with low parasitemia. However, plasma levels of IL‐3 and TGF‐β were lower among participants with high parasitemia than among those with parasite density less than 10000 parasites/µL (Table 5).

**Table 5:** Relationship between inflammatory cytokines and degree of parasitemia among malaria‐infected participants.

Inflammatory cytokines	Malaria parasitemia (p/µL)			*p* Value	Post hoc
	<1000^a^ (*N* = 68)	1000−10,000^b^ (*N* = 17)	>10,000^c^ (*N* = 35)		
*Proinflammatory cytokines*					
TNF‐α (pg/mL)	30.5 (15.0‐43.2)	69.6 (65.0‐71.0)	98.0 (84.0‐105.1)	<.001	x&y, x&z, y&z
IFN‐ɣ (pg/mL)	123.0 (107.0‐137.0)	289.0 (233.5‐296.0)	339.0 (312.2‐362.0)	<.001	x&y, x&z, y&z
IL‐1β (pg/mL)	20.6 (15.0‐27.0)	42.1 (36.3‐51.4)	62.5 (59.7‐66.5)	<.001	x&y, x&z, y&z
IL‐6 (ng/L)	58.1 (39.0‐66.3)	100.4 (98.4‐112.5)	223.0 (124.6‐247.0)	<.001	x&y, x&z, y&z
GM‐CSF (ng/L)	19.3 (15.0‐22.0)	34.0 (30.4‐36.6)	48.1 (39.0‐51.0)	<.001	x&y, x&z, y&z
IL‐3 (pg/mL)	7.5 (6.9‐8.8)	6.7 (6.2‐7.5)	4.2 (3.6‐5.3)	<.001	x&y, x&z, y&z
*Anti‐inflammatory cytokines*					
IL‐10 (ng/L)	64.0 (50.7‐73.0)	195.0 (124.0‐219.3)	401.0 (345.0‐416.3)	<.001	x&y, x&z, y&z
TGF‐β (pg/mL)	2669.0 (2168.0‐3233.0)	1057.0 (991.6‐1267.4)	812.9 (786.0‐955.0)	<.001	x&y, x&z, y&z

*Note*: The data are presented as median (interquartile range), and compared using the Kruskal−Wallis test. *p* < .05 was considered statistically significant.

Abbreviations: g/dL, gram per deciliter; GM‐CSF, granulocyte macrophage‐colony stimulating factor; IFN‐ɣ, interferon‐gamma; IL, interleukin; *N*, number of participants; ng/L, nanogram per liter; p/µL, parasites per microlitre; pg/mL, picogram per milliliter; TGF‐β, transforming growth factor‐beta; TNF‐α, tumor necrosis factor‐alpha.

^a^ Participants with < 1000 parasites/µL;

^b^ Participants with 1000−10,000 parasites/µL;

^c^ Participants with > 10,000 parasites/µL.

### 3.6 Relationship between inflammatory cytokines and anemia severity among malaria‐infected participants

There was a significant relationship between plasma levels of inflammatory cytokines and anemia severity among participants with malaria. Children with severe malarial anemia had higher plasma levels of TNF‐α (*p* < .001), IFN‐ɣ (*p* < .001), IL‐1β (*p* < .001), IL‐6 (*p* < .001), GM‐CSF (*p* < .001), and IL‐10 (*p* < .001) than those with malaria only, and mild/moderate anemia. In contrast, plasma levels of IL‐3 and TGF‐β were relatively lower among participants with severe malarial anemia than those in the uncomplicated malaria group (Table 6).

**Table 6:** Relationship between inflammatory cytokines and anemia severity among malaria‐infected participants.

Inflammatory cytokines	Anemia severity of malaria‐infected participants			*p* Value	Post hoc
	Malaria only^a^ (*N* = 20)	MMA^b^ (*N* = 50)	SMA^c^ (*N* = 50)		
*Proinflammatory cytokines*					
TNF‐α (pg/mL)	14.0 (11.8‐20.6)	41.9 (21.0‐56.6)	86.2 (70.7‐101.1)	<.001	x&y, x&z, y&z
IFN‐ɣ (pg/mL)	75.0 (60.9‐115.0)	132.5 (122.8‐147.3)	317.0 (289.0‐355.3)	<.001	x&y, x&z, y&z
IL‐1β (pg/mL)	12.3 (10.6‐13.7)	25.0 (20.1‐34.2)	59.8 (48.5‐63.6)	<.001	x&y, x&z, y&z
IL‐6 (ng/L)	34.0 (30.0‐42.0)	64.8 (57.0‐70.0)	132.0 (106.3‐238.6)	<.001	x&y, x&z, y&z
GM‐CSF (ng/L)	15.3 (14.0‐20.0)	21.0 (17.4‐24.0)	40.5 (35.5‐49.1)	<.001	x&z, y&z
IL‐3 (pg/mL)	9.6 (8.8‐10.8)	7.0 (6.5‐7.4)	5.3 (3.9‐6.8)	<.001	x&y, x&z, y&z
*Anti‐inflammatory cytokines*					
IL‐10 (ng/L)	51.2 (48.0‐60.0)	69.6 (55.5‐85.3)	356.0 (218.1‐406.6)	<.001	x&y, x&z, y&z
TGF‐β (pg/mL)	3875.4 (3265.9‐4009.0)	2394.5 (1923.8‐2723.3)	923.0 (800.2‐1137.7)	<.001	x&y, x&z, y&z

*Note*: The data are presented as median (interquartile range), and compared using the Kruskal−Wallis test. *p* < .05 was considered statistically significant.

Abbreviations: g/dL, gram per deciliter; GM‐CSF, granulocyte macrophage‐colony stimulating factor; IFN‐ɣ, interferon‐gamma; IL, interleukin; MMA, mild or moderate anemia; N, number of participants; ng/L, nanogram per liter; pg/mL, picogram per milliliter; SMA, severe malarial anemia; TGF‐β, transforming growth factor‐beta; TNF‐α, tumor necrosis factor‐alpha.

^a^ Participants with malaria only;

^b^ Participants with mild or moderate malaria;

^c^ Participants with severe malarial anemia.

### 3.7 Multivariate regression analysis to determine predictors of cytokine levels

Parasite density was observed to be the strongest predictor of all cytokine levels. Parasite density positively associated with IL‐10, GM‐CSF, IL‐6, IL‐1β, IFN‐ɣ, and TNF‐α, but negatively associated with IL‐3 and TGF‐β. In addition, malaria status predicted TNF‐α, IFN‐ɣ, IL‐1β, IL‐6, GM‐CSF and IL‐10 levels, whiles anemia severity predicted only IL‐3, IL‐10 and TGF‐β levels (Table 7).

**Table 7:** Multivariate regression analysis to determine predictors of cytokine levels.

Inflammatory cytokines		Predictor variables				
		Age	Sex	Malaria status	Parasite density	Anemia severity
TNF‐α	*df*	1	1	1	1	1
	S.E.	0.072	5.359	4.155	0.000	4.078
	*β*	.061	−.049	−.671	.830	−.005
	F‐statistic	0.658	0.424	145.421	260.998	0.003
	*p* Value	.418	.516	<.001	<.001	.955
IFN‐ɣ	*df*	1	1	1	1	1
	S.E.	0.217	16.094	13.775	0.000	13.382
	*β*	.028	−.047	−.574	.829	.048
	F‐statistic	0.138	0.388	87.285	258.559	0.270
	*p* Value	.710	.534	<.001	<.001	.604
IL‐1β	*df*	1	1	1	1	1
	SE	0.041	3.046	2.603	0.000	2.543
	*β*	.100	−.020	−.575	.837	.009
	F‐statistic	1.792	0.074	87.781	275.662	0.010
	*p* Value	.182	.787	<.001	<.001	.920
IL‐6	*df*	1	1	1	1	1
	SE	0.152	11.282	9.725	0.000	9.524
	*β*	.032	.062	−.566	.902	.071
	F‐statistic	0.179	0.697	83.978	513.187	0.594
	*p* Value	.673	.405	<.001	<.001	.442
GM‐CSF	*df*	1	1	1	1	1
	SE	0.027	1.991	1.702	0.000	1.632
	*β*	.089	−.070	−.577	.872	.132
	F‐statistic	1.411	0.878	88.821	375.177	2.107
	*p* Value	.236	.350	<.001	<.001	.149
IL‐3	*df*	1	1	1	1	1
	SE	.004	.270	.282	.000	.249
	*β*	−.048	.007	−.006	−.770	.241
	F‐statistic	0.414	0.008	0.006	171.904	7.278
	*p* Value	.521	.931	.936	<.001	.008
IL‐10	*df*	1	1	1	1	1
	SE	0.289	21.452	19.076	0.000	18.389
	*β*	.057	.008	−.524	.917	.189
	F‐statistic	0.571	0.012	67.260	623.964	4.364
	*p* Value	.451	.912	<.001	<.001	.039
TGF‐β	*df*	1	1	1	1	1
	SE	1.932	143.259	150.111	0.003	134.381
	*β*	−.106	.114	−.074	−.709	.235
	F‐statistic	2.015	2.364	0.967	119.110	6.891
	*p* Value	.157	.126	.327	<.001	.010

*Note*: *p* < .05 was considered statistically significant.

Abbreviations: *df*, degree of freedom; GM‐CSF, granulocyte macrophage‐colony stimulating factor; IFN‐ɣ, interferon‐gamma; IL, interleukin; SE, standard error; TGF‐β, transforming growth factor‐beta; TNF‐α, tumor necrosis factor‐alpha.

## 4 DISCUSSION

The equilibrium between proinflammatory and anti‐inflammatory cytokines during *P. falciparum* malaria in children is crucial to prevent life‐threatening complications in disease progression.^12^ This study evaluated selected pro‐ and anti‐inflammatory cytokines among malaria‐infected children in Northern Ghana.

The significantly reduced hemoglobin concentration, RBC count and hematocrit among malaria‐infected children compared to uninfected participants observed in this study is consistent with earlier findings reported in Ghana^15^, ^16^, ^17^ and other developing countries.^10^, ^18^, ^19^ The extensive hemolysis of parasitized and non‐parasitized red cells, extreme sequestration of erythrocytes in organs, direct interaction with plasmodium surface antigens, associated dyserythropoiesis, related complement activation, bone marrow suppression, and the contribution of inflammatory mediators could account for the occurrence of anemia in the malaria‐infected participants.^4^, ^5^, ^9^, ^20^ Thrombocytopenia was observed in children with malaria in this study, and this finding is similar to those of previous studies.^21^, ^22^, ^23^ The reduced platelet count associated with malaria may be due to enhanced sequestration by the spleen, immune‐mediated destruction of thrombocytes and associated ineffective hemopoiesis.^21^, ^23^, ^24^ The reduced lymphocyte count among malaria infected participants observed in this study has also been reported in Ghana, and related it to the associated Fas‐induced apoptosis leading to acute destruction of lymphocytes.^21^ Jiero et al. reported monocytosis in acute malaria as monocytes are involved in phagocytic activities to enhance parasite clearance.^25^ However, the current study observed comparable monocyte counts between malaria infected and uninfected participants. The variation in the outcomes may be due to factors including disease severity, parasitemia, comorbidity and host immunity.^25^

Even though there have been efforts to modify the inflammatory cytokine profile associated with severe malaria in the past via adjunct therapies, this has not yielded the expected clinical outcome. This study found increased plasma levels of proinflammatory cytokines: TNF‐α, IFN‐ɣ, IL‐1β, IL‐6, and GM‐CSF, and the anti‐inflammatory cytokine IL‐10 in malaria‐infected children than the controls without malaria, and the levels were higher in participants with high parasitemia. This finding is consistent with the observations of previous studies.^4^, ^10^, ^12^, ^26^, ^27^, ^28^, ^29^ During the erythrocytic phase of malaria pathogenesis, *P. falciparum*‐erythrocytes express *Pf*EMP‐1 surface antigen which facilitates the adherence of infected erythrocytes to endothelial surfaces by binding to endothelial cell adhesion molecules such as vascular cell adhesion molecule‐1, intercellular adhesion molecule‐1, P‐selectin and E‐selectin.^8^ The *Pf*EMP‐1‐induced extensive sequestration of parasitized and non‐parasitized erythrocytes to endothelial surfaces enhance excessive inflammatory response with subsequent release of proinflammatory cytokines, such as IFN‐ɣ, TNF‐α, IL‐6 and IL‐1β.^8^, ^9^ The enhanced production of proinflammatory cytokines mainly controls the growth of the plasmodium and enhances parasite elimination to inhibit the progression to severe malaria, but unregulated elevation of the cytokines may be detrimental to the host.^6^ Similarly, children with malaria had relatively raised plasma levels of IL‐10, which is comparable with other studies.^4^, ^10^, ^26^, ^28^, ^30^ Interleukin 10 is an anti‐inflammatory and a pleiotropic cytokine that inhibits inflammation by directly reducing the release of proinflammatory cytokines such as TNF‐α and IFN‐ɣ.^31^, ^32^ However, levels of TNF‐α,^10^ IL‐1β,^16^ IL‐6,^8^, ^33^ IFN‐ɣ,^33^ IL‐10,^11^, ^33^ and GM‐CSF^27^ were not different when malaria‐infected children were compared to their counterparts without the infection in previous studies. The difference in the findings remain unclear, but could be linked to factors such as degree of parasitemia, immunity of the host and disease severity.^8^, ^10^, ^11^, ^27^

Although plasma levels of IL‐3 and TGF‐β were similar between children with malaria and the uninfected group, the levels of the cytokines were lower in participants with hyperparasitemia in the present study, and this observation is similar to earlier findings.^32^, ^34^, ^35^ In a study by Meyer et al. that recruited 1015 Ghanaian *P. falciparum*‐infected children at the age of 3 months and followed them up to 2 years, transient increase in IL‐3 was observed among the children with acute *P. falciparum* malaria, but the levels declined as the disease progressed. The IL‐3 single nucleotide polymorphisms rs40401^TT^ and rs40401^CT^ were observed to provide protection against malaria attacks.^35^ Transforming growth factor‐β is a potent anti‐inflammatory and pleiotropic cytokine that inhibits the inflammatory response and regulates malaria pathogenesis, serving as a protective mechanism against severe infection.^36^

In the current study, participants with malaria had higher TNF‐α/IL‐10, TNF‐α/TGF‐β, IFN‐ɣ/TGF‐β, and IL‐1β/TGF‐β, but lower IFN‐ɣ/IL‐10, IL‐1β/IL‐10, and IL‐6/TGF‐β ratios than those in the control group. On the contrary, TNF‐α/IL‐10 and IL‐6/IL‐10 ratios were reduced when malaria‐infected children were compared with uninfected group in Malawi.^37^ Again, a study by Frimpong et al. among Ghanaian children reported that patients with malaria had comparable ratios of IFN‐γ/IL‐10, TNF‐α/IL‐ 10, and IL‐6/IL‐10 than controls without malaria.^11^ Geographical variations may account for the variations in the findings. Whiles the current study was conducted in Northern Ghana, the Frimpong et al. study recruited participants residing in Accra, a coastal area in the country.

In the present study, children with severe malarial anemia had significantly elevated plasma levels of TNF‐α, IFN‐ɣ, IL‐1β, IL‐6, GM‐CSF and IL‐10 than those with uncomplicated malaria, and this is in consonance with earlier findings.^4^, ^26^, ^27^ Even though inflammatory cytokines are essential for inhibiting plasmodium parasite growth and elimination, excessive production negatively affects physiological processes and causes life‐threatening complications such as severe anemia.^6^ TNF‐α has been recognized as a potent inhibitor of erythropoiesis, by retarding erythroid progenitor cell proliferation in humans through the inhibition of erythropoietin synthesis.^38^ In addition, TNF‐α blocks iron recycling from macrophages, restricting iron to storage sites and initiating the development of functional iron deficiency anemia. Again, TNF‐α, independent of hepcidin, blocks the absorption of iron from the duodenum and jejunum by downregulating the expression of divalent metal transporter‐I, increasing the deposition of ferritin in the gastrointestinal tract and reducing iron available for erythropoiesis.^39^ The role of IL‐1β in the development and pathological etiology of anemia has been reported.^40^ Interleukin‐1β negatively influences iron metabolism preventing iron's utilization for hemoglobin synthesis. In addition, IL‐1β suppresses the renal release of erythropoietin, interferes with erythropoietin receptors and eventually promotes early apoptosis of erythroid progenitors.^40^ Despite the effective role of IFN‐ɣ in promoting plasmodium parasite clearance, the proinflammatory cytokine may retard erythropoietic activities by suppressing the proliferation and differentiation of red cell progenitors.^41^ This eventually leads to early apoptosis of erythroblasts with subsequent reduction in red cell parameters in peripheral blood.^9^ The pleiotropic and proinflammatory cytokine, IL‐6 has been identified to passively modulate erythropoiesis.^42^, ^43^, ^44^ IL‐6 induces the release of hepcidin which inhibits the unique cellular iron transporter ferroportin, prevents intestinal iron absorption, interferes with the release of iron from senescent red cells and restricts iron (ferritin) to storage sites. This phenomenon suppresses erythropoiesis through an insufficient supply of iron to the bone marrow and subsequently leads to functional iron deficiency anemia.^43^, ^44^ Independent of iron restriction, IL‐6 has been reported to directly repress erythropoietin‐dependent TF‐1 erythroid maturation.^45^ The contribution of GM‐SCF in the pathogenesis of severe malarial anemia has been emphasized as the proinflammatory cytokine has been reported to inhibit erythropoiesis by interfering with erythroblastic island formation via macrophages, suppressing the proliferation and differentiation of early erythroid progenitors.^46^ On the other hand, children with severe malarial anemia had lower IL‐3 and TGF‐β levels than those with uncomplicated malaria in the current study, and this is similar to previous findings.^33^, ^35^ The protective role of IL‐3 against severe malarial anemia has been reported, as the cytokine principally functions to modulate the production of various blood cells by stimulating the proliferation and differentiation of both early pluripotent stem cells and committed hemoblasts.^47^ IL‐3, in unison with other signaling molecules, such as erythropoietin, IL‐6 and GM‐CSF induces the differentiation of multipotent hemopoietic stem cells into the myeloid progenitor lineage, but in synergy with IL‐7 promotes lymphoid progenitor generation.^48^ TGF‐β is a special protein that critically plays a role in hemopoietic stem cell regulation, with downregulation of its expression in diseases including malaria related to the development of severe anemia.^49^, ^50^

The relative contributions of age, sex, malaria status, parasite density and anemia severity as predictors of cytokine levels were assessed using multivariate linear regression analysis. Parasite density was observed to be the strongest predictor of all cytokine levels. Parasite density positively associated with IL‐10, GM‐CSF, IL‐6, IL‐1β, IFN‐ɣ, and TNF‐α, but negatively associated with IL‐3 and TGF‐β. Previous studies had reported the association between hyperparasitemia and plasma cytokine levels.^27^, ^28^, ^29^ High parasite density is associated with enhanced cytoadherence of both *P. falciparum*‐infected and uninfected erythrocytes to the endothelium. The intense sequestration exacerbates the inflammatory response and cause cytokine storm, especially increasing IL‐10, GM‐CSF, IL‐6, IL‐1β, IFN‐ɣ, and TNF‐α levels, which promotes the progression of severe malaria.^4^, ^8^, ^10^, ^26^, ^27^, ^28^, ^29^ The negative association between parasite density, and IL‐3 and TGF‐β is due to the ability of plasmodium parasites to suppress the release of these two cytokines from immune cells such as monocytes, CD4+ and CD8+ cells.^34^

### 4.1 Major strength and limitations

This study revealed the relationship between severe malarial anemia and inflammatory cytokines in *P. falciparum*‐infected children. Findings from the current study were restricted to children aged 1‐12 years. In addition, the study could not assess other important pro‐ and anti‐inflammatory cytokines. Also, the study could not perform molecular assessment to identify sub‐microscopic malaria infection.

## 5 CONCLUSION

Malaria presents with enhanced release of TNF‐α, IFN‐ɣ, IL‐1β, IL‐6, GM‐CSF, and IL‐10, but downregulates IL‐3 and TGF‐β in Ghanaian children. Inflammatory cytokines may contribute to the development of severe malarial anemia in children. However, IL‐3 and TGF‐β may offer protection against severe malarial anemia and prevent further complications. Therapeutic protocols should target pro‐ and anti‐inflammatory cytokines for the management of severe malarial anemia in children. Future genotypic studies on cytokines in malaria are recommended.

## AUTHOR CONTRIBUTIONS

**Charles Nkansah:** Conceptualization, methodology, investigation, formal analysis, validation, visualization, writing—original draft, writing—review and editing, supervision. **Felix Osei‐Boakye:** Validation, methodology, writing—original draft, writing—review and editing. **Gabriel Abbam:** Methodology, writing—original draft, writing—review and editing. **Samuel K. Appiah:** Validation, methodology, writing—original draft, writing—review and editing. **Samira Daud:** Validation, methodology, writing—original draft, writing—review and editing. **Bright Boakye:** Conceptualization, methodology, resources, investigation, writing—original draft, writing—review and editing. **Samsiyatu Abdulai:** Conceptualization, methodology, resources, investigation, writing—original draft, writing—review and editing. **Madina Ahmed:** Conceptualization, methodology, resources, investigation, writing—original draft, writing—review and editing. **Theophilus B. Antwi**: Conceptualization, methodology, resources, investigation, writing—original draft, writing—review and editing. **Birago Boateng:** Conceptualization, methodology, resources, investigation, writing—original draft, writing—review and editing. **Miigbat P. Libatin:** Conceptualization, methodology, resources, investigation, writing—original draft, writing—review and editing. **Alexander S. Mensah:** Conceptualization, methodology, resources, investigation, writing—original draft, writing—review and editing. **Mary K. Missah:** Conceptualization, methodology, resources, investigation, writing—original draft, writing—review and editing. **Richard V. Duneeh:** Validation, methodology, writing—original draft, writing—review and editing. **Ashiya Haruna:** Conceptualization, methodology, resources, investigation, writing—original draft, writing—review and editing. **Stephany Adda:** Conceptualization, methodology, resources, investigation, writing—original draft, writing—review and editing. **Pagnaa G. Abdul‐Rauf:** Conceptualization, methodology, resources, investigation, writing—original draft, writing—review and editing. **Zacharia A. Ofori:** Conceptualization, methodology, resources, investigation, writing—original draft, writing—review and editing. **George B. Fosu:** Conceptualization, methodology, resources, investigation, writing—original draft, writing—review and editing. **Sandra Segnitome:** Conceptualization, methodology, resources, investigation, writing—original draft, writing—review and editing. **Isaac Adjei:** Data curation, methodology, investigation, writing—original draft, writing—review and editing. **Emmanuel Appiah‐Kubi:** Methodology, investigation, formal analysis, writing—original draft, writing—review and editing. **Moses Banyeh:** Methodology, visualization, validation, writing—original draft, writing—review and editing. **Charles A. Derigubah:** Visualization, validation, writing—original draft, writing—review and editing. **Muniru M. Tanko:** Investigation, writing—original draft, writing—review and editing. **Ejike F. Chukwurah:** Conceptualization, methodology, validation, visualization, writing—original draft, writing—review and editing, supervision.

## CONFLICT OF INTEREST STATEMENT

The authors declare no conflict of interest.

## ETHICS STATEMENT

This study was conducted in accordance with the Declaration of Helsinki on Research Involving Humans. The Institutional Review Board of University for Development Studies, Ghana approved this study (UDS/IRB/15/2023). Permission was gotten from Tamale Teaching Hospital. Written informed consent was obtained from the parents or guardians of the participants by signing or thumb‐printing the written informed consent form.

## Data Availability

The data supporting findings in this study are available from the corresponding author upon reasonable request.
